# A Case of Drug-Induced Bullous Pemphigoid With an Isomorphic Response and Updated Review of Koebnerization in Bullous Diseases

**DOI:** 10.7759/cureus.20647

**Published:** 2021-12-23

**Authors:** Austin B Ambur, Rajiv Nathoo, Sadia Saeed

**Affiliations:** 1 Dermatology, Kansas City University-Graduate Medical Education Consortium/Advanced Dermatology and Cosmetic Surgery, Oviedo, USA; 2 Dermatopathology, Kansas City University-Graduate Medical Education Consortium/Advanced Dermatology and Cosmetic Surgery, Oviedo, USA

**Keywords:** isomorphic reaction, koebner phenomenon, pemphigoid, medical dermatology, general dermatology, bullous pemphigoid, bullous dermatoses

## Abstract

Bullous pemphigoid (BP) can be a challenging diagnosis as it can mimic a variety of other inflammatory conditions. An isomorphic response may be seen in a variety of cutaneous diseases; however, there is a paucity of reports associated with BP. We present a case of recurrent drug-induced BP with an isomorphic response to trauma in a 73-year-old Caucasian male. Bumetanide was determined to be the inciting cause of the initial disease. The patient was initially treated with prednisone tapers and topical steroids. Mycophenolate Mofetil was eventually started after numerous flares. He later developed isomorphic bullous lesions at the periphery of a skin graft that was completed following a traumatic fall. He was started on clobetasol ointment with full resolution over the following month.

## Introduction

Bullous pemphigoid (BP) is an autoimmune blistering disease characterized by tense subepithelial blisters arising from normal-to-erythematous skin in a confined distribution. The Koebner phenomenon (KP) was first described in psoriasis patients with the appearance of new psoriasiform lesions on uninvolved skin after trauma. The definition has been broadened after recognizing that many cutaneous diseases arise after trauma to the skin and now may be referred to as an isomorphic response. KP may be classified into several different groups including true koebnerization, pseudo-koebnerization, and localized trauma-induced koebnerization. KP is associated with many skin conditions, including vitiligo, lichen planus, Darier disease, psoriasis, and is rarely reported in autoimmune bullous dermatosis [1]. An isomorphic reaction has not been established with drug-induced bullous pemphigoid. It is important to recognize the various bullous dermatoses associated with an isomorphic reaction to make the correct diagnosis and expedite the appropriate treatment. We report a case of BP exhibiting an isomorphic response and explore the possible mechanisms behind this process. We also use this case to expand upon the differential diagnosis of koebnerization as it relates to bullous diseases.

## Case presentation

**Figure 1:**
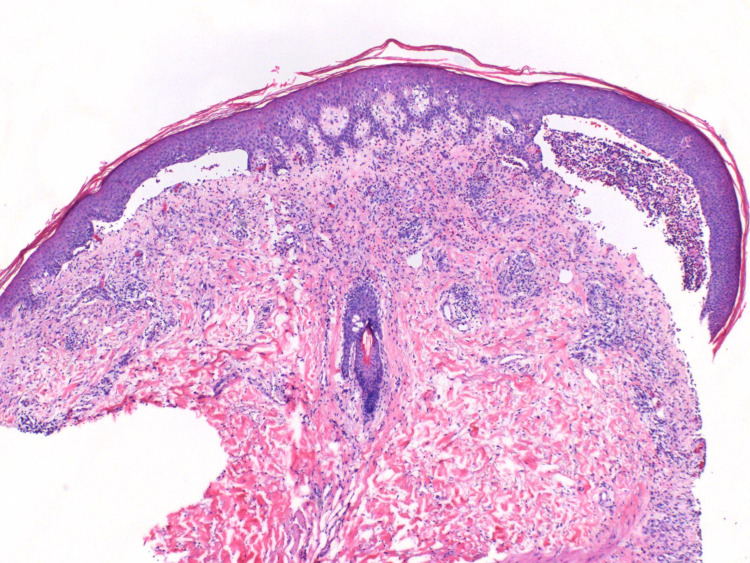
Hematoxylin and eosin (H&E) stain 4x original biopsy showing a subepidermal blister with an eosinophil-rich infiltrate.

**Figure 2:**
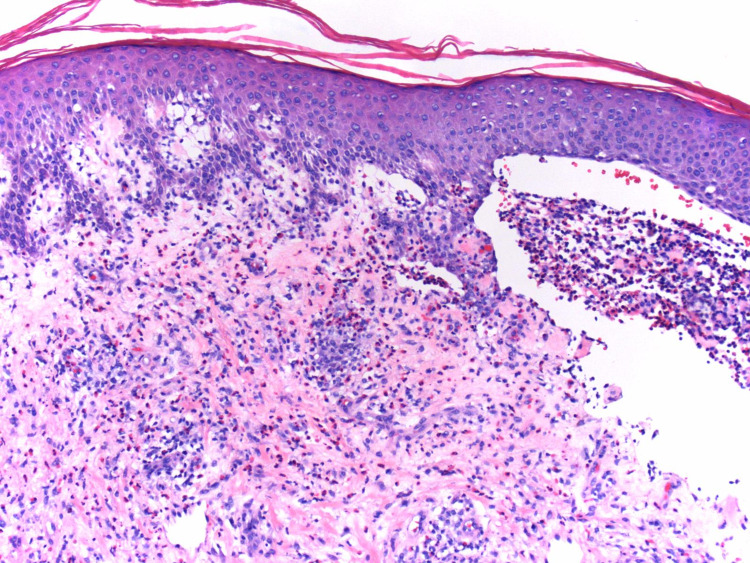
Hematoxylin and eosin (H&E) stain 10x original biopsy highlighting the abundance of eosinophils associated with a subepidermal blister.

A 73-year-old Caucasian male with a history of heart failure presented with a tense bullous eruption of two weeks in duration. The eruption was localized to the bilateral proximal arms, legs, and trunk. Punch biopsy was performed and submitted for both routine microscopy and direct immunofluorescence analysis (DIF). The specimens demonstrated subepidermal bulla formation with eosinophils (Figures 1-2) and linear IgG and C3 along the dermal-epidermal junction via DIF.

**Figure 3:**
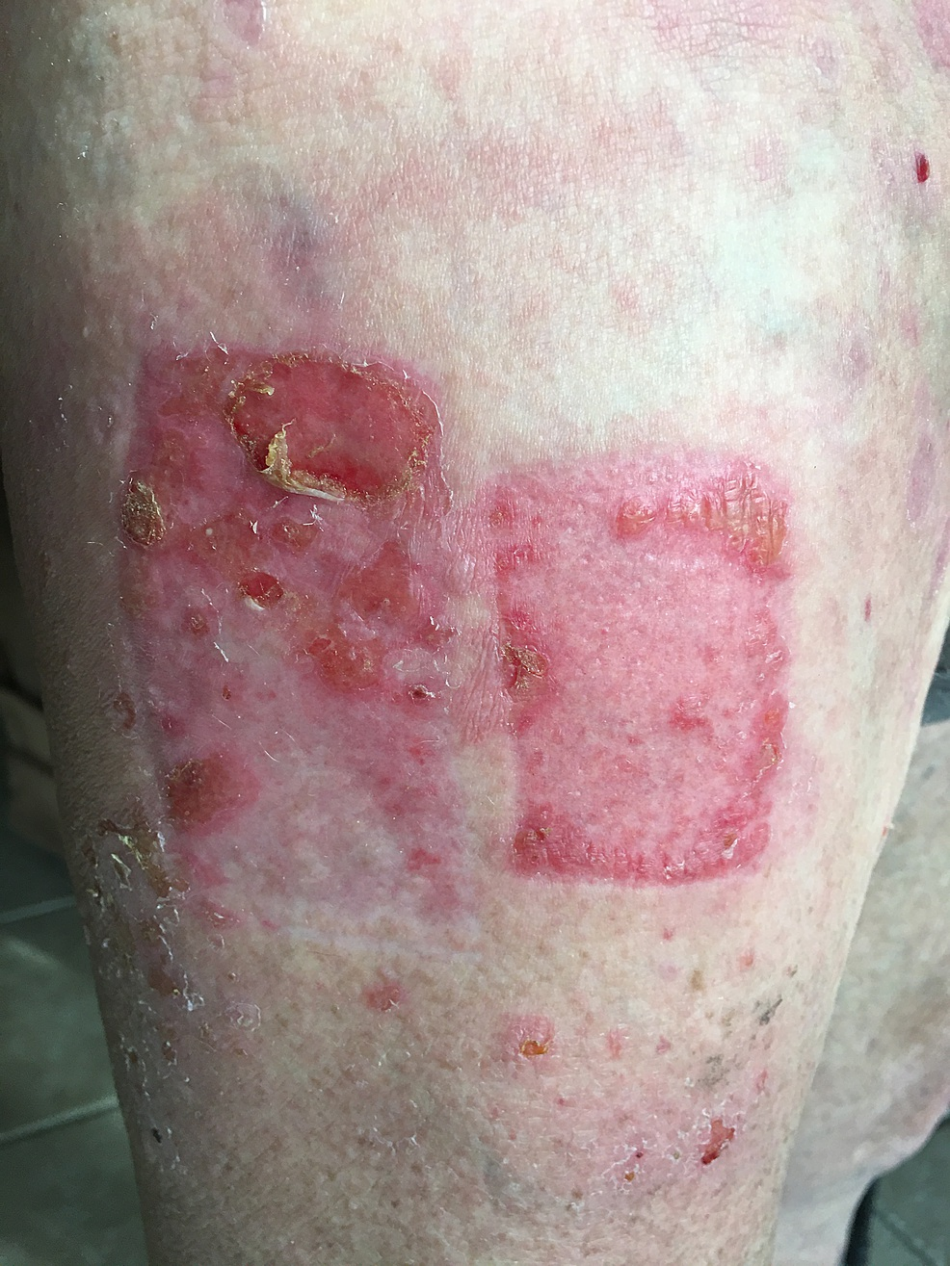
Several tense bullae and erosions localized to the periphery of the skin graft donor site.

Enzyme-linked immunosorbent assay (ELISA) revealed elevations in BP180 and BP230 antibodies. The histopathologic and immunophenotypic features supported a diagnosis of BP. Bumetanide was recently started for heart failure with worsening lower extremity edema and dyspnea. Medication reconciliation was unremarkable for any additional sources or drug-drug interactions. Bumetanide was held and the patient began a prednisone taper starting at 60 mg daily and doxycycline, 100 mg twice daily. The patient had subsequent flares and was ultimately started on Mycophenolate Mofetil with marked improvement over the following months. The patient’s course was complicated by a traumatic fall requiring split-thickness skin grafting on the right thigh. On follow-up, he was noted to have multiple tense bullae localized to the periphery of the donor site (Figure 3).

**Figure 4:**
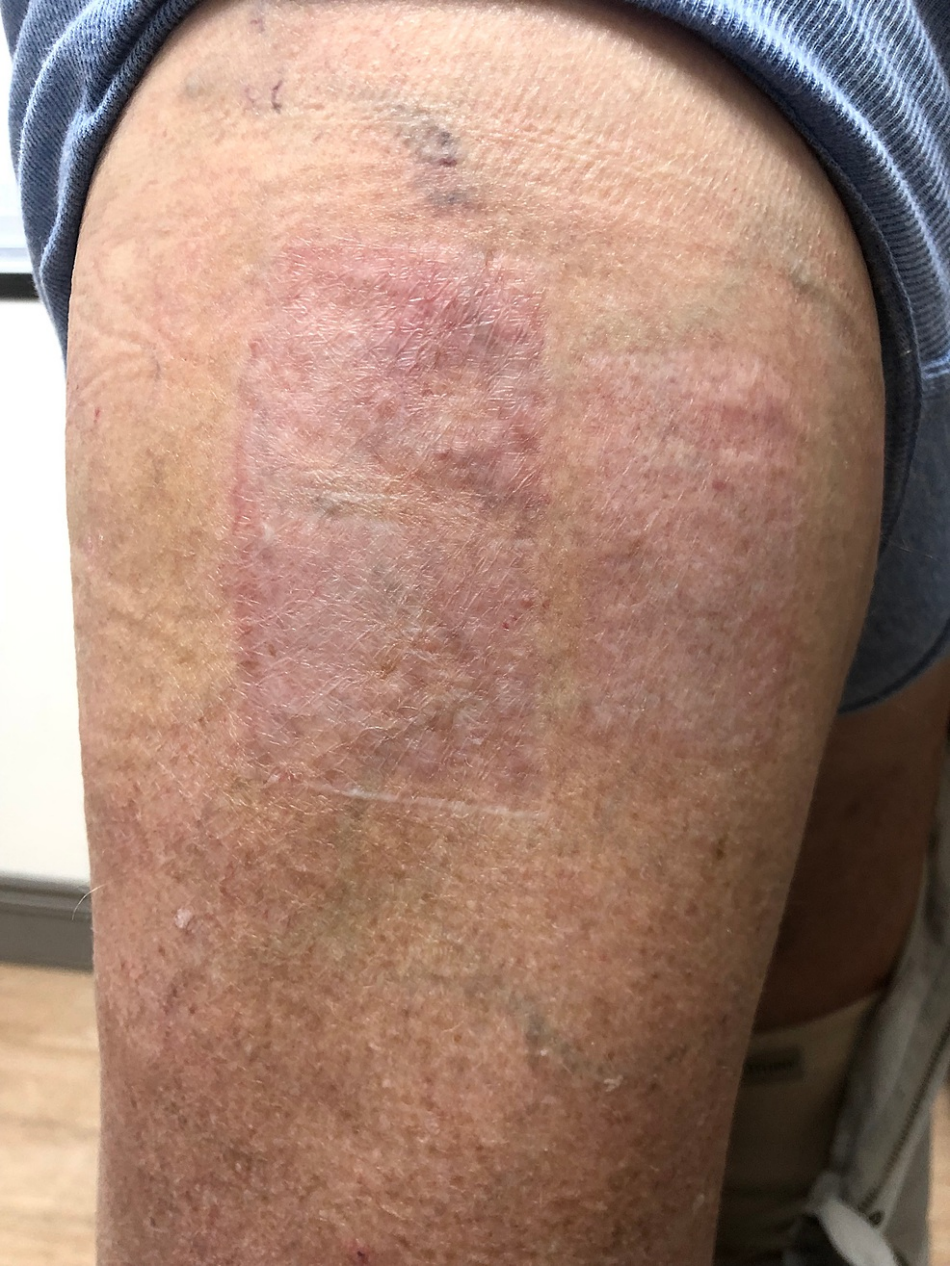
Well-healed skin graft donor site with resolution of bullous lesions.

The remaining skin examination was unremarkable for additional active BP lesions. No new medications had been initiated. The newly formed bullous lesions were attributed to an isomorphic response to trauma from the skin graft. He was then started on clobetasol 0.05% ointment with complete resolution of lesions over the following month (Figure 4).

## Discussion

KP is an isomorphic reaction characterized by the appearance of new lesions on previously unaffected skin in response to cutaneous injury. The pathogenic mechanism is not well-established; however, it may involve cytokines, stress proteins, adhesion molecules, and autoantigens. KP may be classified into several different groups. True koebnerization refers to processes that are reproducible by any manner of cutaneous injury and is seen in vitiligo, psoriasis, and lichen planus. Pseudo-koebnerization occurs by seeding of infectious agents into traumatized skin and is seen with molluscum contagiosum, verrucae, and pyoderma gangrenosum [1]. Localized trauma-induced koebnerization may occur in Darier disease, erythema multiforme, and granuloma annulare. Koebnerization may be observed in a number of autoimmune blistering diseases including BP. Although trauma may be an inciting factor for BP, there are no reported cases of an isomorphic response associated specifically with drug induced-BP. The cause is largely unknown; however, several theories exist that may explain the pathogenesis. The leading theory for koebnerization seen in bullous diseases has been described as secondary to epitope spreading. Epidermal injury or inflammation is thought to uncover the antigen in predisposed individuals and result in new koebnerization lesions [2]. Antibodies to the BP180 transmembrane protein are the primary mediators of BP. The intracellular location of BP230 hemidesmosomal plaque presumably makes it less prone to autoantibody attack. Autoantibodies directed against BP230 may result as a secondary phenomenon that is important in the pathogenesis of epitope spreading. KP has been reported to only occur if there are both epidermal injury and dermal inflammation [1]. The isomorphic response observed in our patient was attributed to the epidermal injury caused by the skin graft paired with the dermal inflammation caused by bumetanide. The diagnosis can be challenging, as it can mimic other vesiculobullous diseases including pemphigus vulgaris, bullous lichen planus, linear IgA bullous dermatosis, dermatitis herpetiformis, erythema multiforme, and Sweet’s syndrome [3-9]. We provide an updated list of koebnerization in bullous diseases, along with the inciting dermatoses, which will be useful to the practicing physician (Table 1).

**Table 1:** Koebnerization in bullous diseases and their inciting dermatoses. UVB: ultraviolet B

Koebnerization in Bullous Diseases	Inciting Dermatosis
Bullous pemphigoid	Burns, phototherapy, x-ray irradiation, surgical incision sites, venous stasis, lymphedema, fistulas, ostomies
Pemphigus vulgaris	Hair transplant, Hijama (wet cupping), burn scar, Mantoux test, surgical wound
Bullous lichen planus	Not specifically stated
Linear IgA bullous dermatosis	Injection site reaction, friction
Mucous membrane pemphigoid	Ill-fitting dentures
Dermatitis herpetiformis	Psoriasis
Erythema multiforme	Folliculitis, laceration from human bite, scratch, surgical scar, traumatized nail fold, UVB
Sweet’s syndrome	Nonspecific irritation and inflammation of the skin

## Conclusions

Although a number of autoimmune blistering diseases have been associated with an isomorphic response, there are no reports specifically associated with drug-induced BP. This case highlights a unique phenotypical characteristic of drug-induced BP that has been rarely described. This article reviews the proposed pathogenic mechanisms behind various conditions linked to koebnerization. It also expands upon the differential diagnosis of koebnerization as it relates to bullous diseases. The expanded list will aid in the diagnosis of koebnerized bullous diseases and help clinicians provide appropriate counseling to patients.

## References

[1] Rubin AI, Stiller MJ (2002). A listing of skin conditions exhibiting the koebner and pseudo-koebner phenomena with eliciting stimuli. J Cutan Med Surg.

[2] Rotunda AM, Bhupathy AR, Dye R, Soriano TT (2005). Pemphigus foliaceus masquerading as postoperative wound infection: report of a case and review of the Koebner and related phenomenon following surgical procedures. Dermatol Surg.

[3] Mai Y, Nishie W, Sato K (2018). Bullous pemphigoid triggered by thermal burn under medication with a dipeptidyl peptidase-iv inhibitor: a case report and review of the literature. Front Immunol.

[4] Algoet C, Mostinckx S, Theate I, Vanhooteghem O (2020). Localized bullous pemphigoid: four clinical cases and a literature review. Clin Case Rep.

[5] Balighi K, Daneshpazhooh M, Azizpour A, Lajevardi V, Mohammadi F, Chams-Davatchi C (2016). Koebner phenomenon in pemphigus vulgaris patients. JAAD Case Rep.

[6] Verma R, Vasudevan B, Kinra P, Vijendran P, Badad A, Singh V (2014). Bullous lichen planus. Indian J Dermatol Venereol Leprol.

[7] Rashid Dar N, Raza N (2008). Drug induced linear IgA disease with unusual features: Koebner phenomenon, local insulin sensitivity and annular blister of the nipples. Acta Dermatovenerol Croat.

[8] Lee R, Lobo Y, Spelman L (2021). Development of dermatitis herpetiformis in chronic plaque psoriasis. Case Rep Dermatol.

[9] Shi CR, Charrow A, Granter SR, Christakis A, Wei EX (2018). Unilateral, localized bullous pemphigoid in a patient with chronic venous stasis. JAAD Case Rep.

